# Early nurse-assessed intrinsic capacity stratifies rehospitalization risk after percutaneous coronary intervention in coronary artery disease patients

**DOI:** 10.3389/fcvm.2026.1763248

**Published:** 2026-04-20

**Authors:** Yuning Zhao, Wenwen Zhang, Jing Yang, Yuqi Fang, Yizhu Yan, Guangyao Zhai

**Affiliations:** Cardiology Department, Beijing Luhe Hospital Affiliated to Capital Medical University, Beijing, China

**Keywords:** coronary artery disease, intrinsic capacity, nurse, PCI, rehospitalization

## Abstract

**Background:**

Intrinsic capacity (IC) has shown potential in predicting health outcomes in older adults. However, its prognostic value in patients with coronary artery disease (CAD) following percutaneous coronary intervention (PCI) has not been established.

**Methods:**

In this retrospective cohort study, patients with CAD undergoing PCI were included. IC score was assessed within 48 h of admission using a structured nurse-administered questionnaire. The primary outcome was all-cause rehospitalization. Secondary outcomes included cardiovascular rehospitalization and non-cardiovascular rehospitalization. Kaplan–Meier analysis, Cox proportional hazards models, and restricted cubic spline (RCS) were used to estimate the relation between IC score and rehospitalization. Subgroup analysis and receiver operating characteristic (ROC) curves were used to assess predictive performance.

**Results:**

A higher IC score, indicating poorer IC, was independently associated with increased all-cause rehospitalization risk (HR = 3.07 for IC = 4 compared with IC = 0, 95% CI 1.89–5.00) and cardiovascular rehospitalization risk (HR = 5.23 for IC = 4 compared with IC = 0, 95% CI 2.30–11.89). Subgroup analyses showed that this relationship remained consistent across lesion morphologies and revascularization strategies. In contrast, IC score was not a significant predictor of non-cardiovascular rehospitalization. RCS curves showed the linear positive relationship between IC score and HR of cardiac rehospitalization with the cutoff of 2.5. ROC curve for all-cause rehospitalization showed IC score with the AUC of 0.692 (95% CI: 0.664–0.729).

**Conclusion:**

IC score is an accessible, independent, and robust predictor of cardiovascular rehospitalization after PCI in CAD patients.

## Introduction

Coronary artery disease (CAD) continues to impose a substantial burden on patients and healthcare systems (1). Hospital readmission following percutaneous coronary intervention (PCI) is common and related to worse long-term prognosis. Studies have reported that over 40% are readmitted within one year due to cardiovascular or non-cardiovascular complications (2). These recurrent hospitalizations not only signal an unfavorable prognosis but also significantly impair patients' quality of life (3). Thus, identifying individuals at risk of readmission and implementing early interventions are crucial for improving patient-centered outcomes.

As a chronic condition, CAD requires long-term disease nursing, lifestyle management, and treatment adherence (4). In this context, an individual's capacity to manage their health and daily life becomes a pivotal factor in long-term prognosis (5). Recently, the concept of intrinsic capacity (IC) has gained increasing attention, which reflects an individual's physical and mental reserves (6). Introduced by the World Health Organization in 2015, IC shifts the focus of care from a disease-based to a function-oriented model. IC encompasses multiple domains, such as locomotion, sensory function, vitality, and psychological status, offering a new view of an individual's overall functional capacity (7). It is now considered a promising tool for clinical risk stratification.

Emerging evidence has linked lower IC to adverse outcomes in various clinical populations. A recent meta-analysis involving over 40,000 older adults revealed that declines in IC are strongly associated with functional deterioration and mortality, regardless of baseline health status (8, 9). Additionally, in hospitalized older adults, reduced IC has been independently associated with the presence of sarcopenia and increased frailty risk (10). In oncology, Maheshwari et al. found that IC level could predict survival among older patients with gastrointestinal cancers, further supporting the utility of IC in risk prediction across a wide range of diseases and care settings (11).

Within cardiovascular research, interest in IC is rapidly growing. Several recent cohort studies have shown that lower IC or unfavorable IC trajectories are associated with higher incidence of cardiovascular events and mortality. In a large prospective study using UK Biobank data, Ramírez-Vélez et al. found that lower IC scores predicted both the onset and fatality of cardiovascular disease over a median follow-up of 11 years (12). Similarly, a nationwide cohort from China demonstrated that deteriorating IC trajectories significantly elevated the risk of new-onset cardiovascular disorders in community-dwelling older adults (13). Other work has confirmed that in elderly patients with established cardiovascular disease, lower IC is closely tied to faster functional decline, poorer self-management ability, and higher medical utilization (14).

However, current research on IC has primarily focused on its association with the onset of cardiovascular disease or its predictive value for mortality in general populations. Far less is known about the prognostic utility of IC in individuals with established CAD, especially those who have undergone PCI, which has been a widely used and effective treatment. Given that PCI-treated patients represent a growing and heterogeneous population, hospital readmission remains a key quality indicator and a frequent, often preventable, adverse outcome (15). Yet, no research to date has explored whether IC can serve as an independent predictor of rehospitalization in this specific patient group. Considering the increasing importance of functional health in long-term management, assessing the relationship between IC and post-PCI readmission risk is both necessary and clinically meaningful.

Therefore, the aim of this study was to investigate the association between IC and hospital readmission in patients with CAD undergoing PCI. By doing so, we aim to provide new insights into personalized risk stratification and long-term care planning in CAD populations.

## Methods

### Study design

This study was a retrospective observational cohort study. Our institutional ethics committee approved this study, and the requirement for informed consent was waived.

### Study population

**Figure 1:**
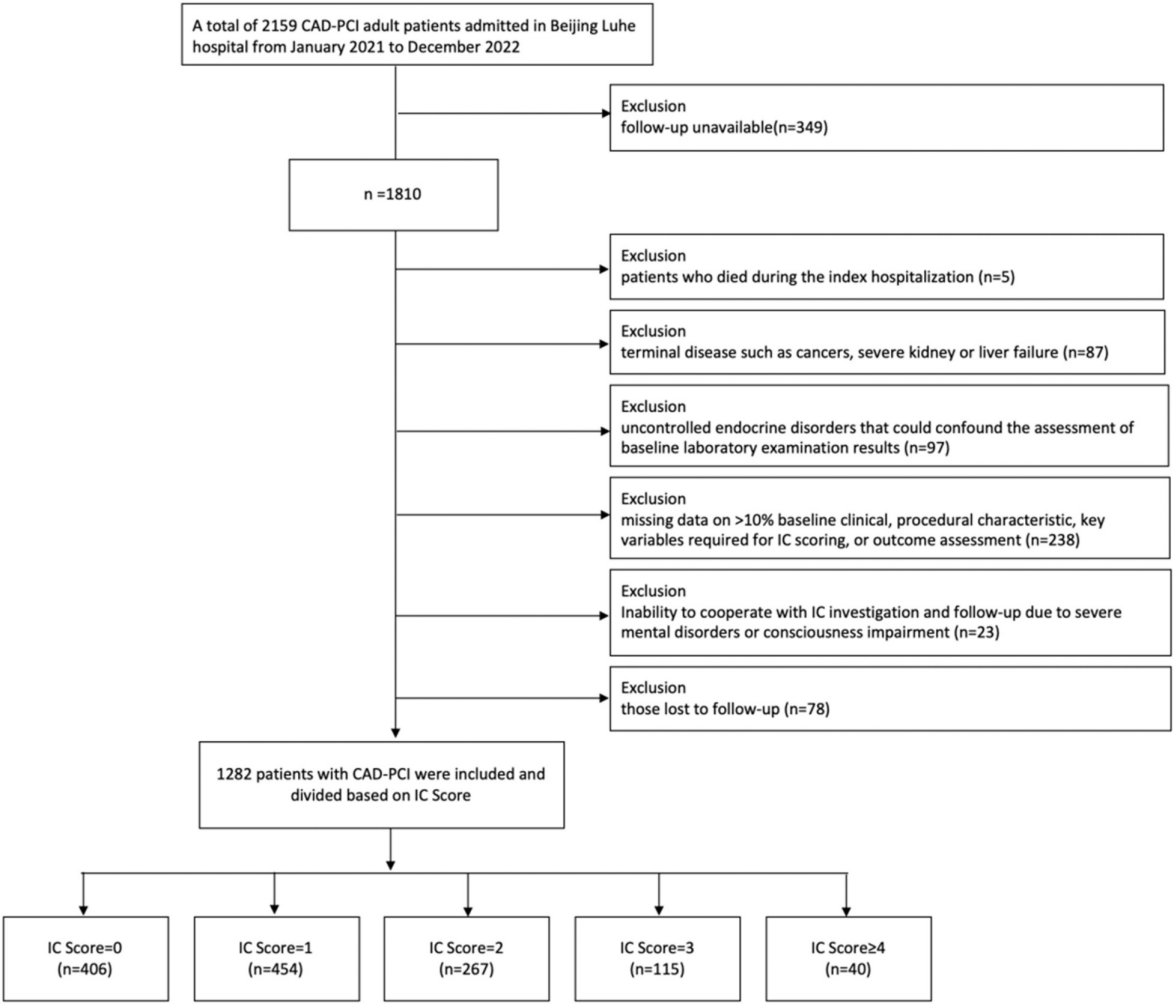
Study flowchart. IC, intrinsic capacity; CAD, coronary artery disease; PCI, percutaneous coronary intervention.

The study collected patients with CAD who underwent PCI at the department of cardiology of Beijing Luhe Hospital between January 2021 and December 2022. Eligible CAD patients were based on current clinical guidelines (16). Inclusion criteria were: (1) were aged 18 years or older and willing to cooperate with follow-up; (2) had a confirmed diagnosis of CAD and underwent PCI during the hospitalization. Exclusion criteria were: (1) patients who died during the index hospitalization; (2) those lost to follow-up; (3) patients with missing data on >10% baseline clinical, procedural characteristics, key variables required for IC scoring, or outcome assessment; (4) severe mental illness or disturbances of consciousness rendered patients unable to participate in IC surveys and follow-up work; (5) terminal disease such as cancers, severe kidney or liver failure; (6) patients with uncontrolled endocrine disorders (e.g., poorly controlled thyroid dysfunction, or other hormone-related conditions) that could confound the assessment of baseline laboratory examination results (Figure 1).

### Definitions

IC was assessed using a structured questionnaire encompassing multiple domains, including the psychological domain (exhaustion and sleep duration), the sensory domain (vision and hearing), the vitality domain (grip strength, weight, and walking).

Each impaired domain was assigned a score of 1, and the total IC score ranged from 0 to 7. A higher IC score indicates greater functional impairment and therefore lower IC. The full questionnaire and scoring criteria are provided in the Supplementary Materials. PCI-Related definitions followed the current guidelines or consensus statements which were assessed by two experienced interventional cardiologists blinded to patient outcomes. The detailed definitions on coronary angiographic morphology are in the Supplementary Materials (17).

### Data collection

Data collection was conducted systematically during the hospitalization. IC was assessed within 48 h of admission by a trained nurse using a structured questionnaire. To ensure accuracy, the assessment was independently reviewed and verified by a second nurse. Vital signs and the clinical classification of CAD were recorded upon admission. Information on past history and comorbidities was extracted from the patients' electronic medical records. Laboratory test results were obtained within the first 48 h of admission, including hematologic, biochemical, and inflammatory markers. Data related to PCI, including lesion characteristics and procedural details, were obtained during coronary angiography and PCI. These angiographic features were independently evaluated by two interventional cardiologists, both of whom were board-certified and blinded to patient outcomes.

### Outcomes measurement

The primary outcome of this study was all-cause rehospitalization during the follow-up period. Secondary outcomes included cardiac rehospitalization and non-cardiac rehospitalization, which were analyzed separately. All rehospitalization events were identified through a combination of electronic medical record review and telephone follow-up. The follow-up period was up to 36 months. Cardiac rehospitalization was defined as hospital admission due to cardiovascular causes, including recurrent angina, myocardial infarction, heart failure, arrhythmia, or any unplanned coronary revascularization procedure. Non-cardiac rehospitalization referred to admissions resulting from other systemic conditions not primarily related to cardiac pathology. All outcome events were independently adjudicated by two trained nurses who were blinded to patients' IC scores. Discrepancies were resolved by consensus or by consultation with a third senior doctor. Patients who experienced unexplained out-of-hospital death during follow-up were not included in the rehospitalization outcome analysis.

### Statistical analysis

Statistical analyses included descriptive statistics, group comparisons, Cox regression, Kaplan–Meier survival analysis, restricted cubic spline modeling, subgroup analyses with forest plots, and Receiver operating characteristic (ROC) curve evaluation. Analyses were conducted using STATA 17 and R (v4.2.1). Detailed methods and packages are provided in the Supplementary Materials.

## Results

The total 1,282 participants had an average age of 62.2 years (±11.2), with 74.7% of participants being male. Among them, 31.67% (406 participants) have an IC score of 0, 35.41% (454 participants) have a score of 1, 20.83% (267 participants) have a score of 2, 8.97% (115 participants) have a score of 3, and 3.12% (40 participants) have a score of 4. When comparing the rehospitalization and non-rehospitalization groups, several significant differences were observed. The rehospitalization group was older (*P* < 0.001) and had a higher average weight (*P* = 0.045) and BMI (*P* = 0.007). Additionally, the rehospitalization group showed higher levels of monocytes (*P* < 0.001), platelets (*P* = 0.043), uric acid (*P* = 0.038), and triglyceride (*P* = 0.040) along with a lower albumin level (*P* = 0.046). In terms of medication use, the rehospitalization group had a higher proportion of patients using ARBs (*P* = 0.009) and a lower proportion using sacubitril valsartan (*P* = 0.022). No significant differences were found in other variables between the two groups (Table 1). Next, we summarized the difference of IC score and its components between the rehospitalization and non-rehospitalization groups. The rehospitalization group showed a higher median IC score compared to the non-rehospitalization group (*P* < 0.001). No significant differences were found in the psychological domain. In the sensory domain, the rehospitalization group had a higher prevalence of vision impairment (*P* < 0.001) and hearing difficulty (*P* < 0.001) compared to the non-rehospitalization group. In the vitality domain, the rehospitalization group had a higher proportion of individuals with declined grip strength (*P* = 0.003). No significant difference was observed for weight loss or walking difficulty between the groups (Table 2). Building on the previous analysis of rehospitalization status, we now compare the data based on gender. In the total population, the median IC score was 1.0 for both males and females (*P* = 0.214). Regarding all the components of IC score, no significant differences were found between males and females (Table 3).

**Table 1:** Clinical characteristics baseline.

Variables	Total	Non-rehospitalization	Rehospitalization	*P*
Demographic characteristics				
Age, mean (±SD), yrs	62.2 ± 11.2	59.1 ± 10.3	70.3 ± 9.1	<0.001
Male, *N* (%)	958 (74.7%)	695 (75.1%)	263 (73.7%)	0.639
SBP, mean (±SD), mmHg	126.5 ± 18.5	126.3 ± 18.7	126.8 ± 18.1	0.693
DBP, mean (±SD), mmHg	73.8 ± 12.3	73.7 ± 12.5	74.2 ± 11.7	0.456
Pulse, mean (±SD), bpm	72.7 ± 10.5	72.4 ± 10.6	73.5 ± 10.3	0.092
Weight, mean (±SD), kg	73.4 ± 13.2	72.9 ± 13.1	74.7 ± 13.5	0.045
Height, mean (±SD), cm	167.7 ± 8.0	167.8 ± 8.0	167.7 ± 8.1	0.981
BMI, mean (±SD), kg/m^2^	25.9 ± 3.6	25.8 ± 3.5	26.4 ± 3.8	0.007
Comorbidities				
DM, *N* (%)	565 (44.1%)	404 (43.7%)	161 (45.1%)	0.691
Hypertension, *N* (%)	855 (66.7%)	626 (67.7%)	229 (64.1%)	0.256
Hypercholesterolemia, *N* (%)	976 (76.1%)	700 (75.7%)	276 (77.3%)	0.587
Atrial fibrillation, *N* (%)	77 (6.0%)	52 (5.6%)	25 (7.0%)	0.423
Laboratory examination				
WBC, mean (±SD), ×10^9^/L	7.7 ± 2.2	7.6 ± 2.1	7.9 ± 2.5	0.055
RBC, mean (±SD), ×10^9^/L	4.4 ± 0.6	4.4 ± 0.6	4.4 ± 0.6	0.916
Hemoglobin, mean (±SD), g/L	135.0 ± 19.2	135.1 ± 19.2	135.0 ± 19.3	0.931
Lymphocytes, mean (±SD), ×10^9^/L	1.8 ± 0.6	1.8 ± 0.6	1.8 ± 0.6	0.702
Monocytes, mean (±SD), ×10^9^/L	0.5 ± 0.2	0.5 ± 0.2	0.5 ± 0.2	<0.001
Neutrophils, mean (±SD), ×10^9^/L	5.3 ± 2.0	5.2 ± 2.0	5.4 ± 2.3	0.103
Platelet, Median [IQR], ×10^9^/L	212.0 [180.0;254.0]	210.0 [179.0;252.0]	222.0 [182.0;263.0]	0.043
Albumin, mean (±SD), g/L	41.3 ± 3.9	41.5 ± 4.0	41.0 ± 3.9	0.046
Total bilirubin, mean (±SD), μmol/L	13.4 ± 8.0	13.5 ± 8.3	13.2 ± 6.9	0.591
Direct bilirubin, mean (±SD), μmol/L	4.3 ± 3.1	4.4 ± 3.4	4.2 ± 2.2	0.397
ALT, Median [IQR], U/L	22.0 [15.0;34.0]	22.0 [14.0;33.0]	23.0 [15.0;38.0]	0.133
AST, Median [IQR], U/L	22.0 [17.0;33.0]	21.0 [16.0;32.0]	22.0 [17.0;35.0]	0.120
ALP, Median [IQR], U/L	77.0 [64.0;91.0]	77.0 [64.0;91.0]	78.0 [64.2;92.0]	0.680
Glucose, mean (±SD), mmol/L	7.2 ± 3.0	7.2 ± 3.0	7.3 ± 3.0	0.396
HbA1c, mean (±SD), %	6.8 ± 1.3	6.8 ± 1.3	6.8 ± 1.3	0.882
Uric acid, Median [IQR], μmol/L	361.0 [299.6;430.6]	356.3 [295.0;429.6]	363.2 [313.7;438.2]	0.038
Urea, Median [IQR], mmol/L	5.9 [4.8;7.6]	5.9 [4.8;7.5]	6.0 [4.8;7.6]	0.907
Creatinine, Median [IQR], μmol/L	77.4 [66.1;91.7]	77.2 [66.1;90.7]	77.9 [66.2;93.8]	0.644
EGFR, Median [IQR]	87.3 [70.7;99.6]	87.7 [71.0;100.1]	86.2 [68.6;98.8]	0.560
Triglyceride, mean (±SD), mmol/L	1.8 ± 1.2	1.7 ± 1.1	1.9 ± 1.3	0.040
TC, mean (±SD), mmol/L	4.0 ± 1.1	4.0 ± 1.1	4.1 ± 1.1	0.083
LDL-c, mean (±SD), mmol/L	2.4 ± 0.9	2.3 ± 0.9	2.4 ± 0.9	0.103
HDL-c, mean (±SD), mmol/L	1.0 ± 0.2	1.0 ± 0.2	1.0 ± 0.2	0.088
hs-CRP, Median [IQR], U/L	2.1 [0.8;6.9]	2.0 [0.8;6.6]	2.2 [0.8;8.0]	0.385
Homocysteine, Median [IQR], μmol/L	14.8 [11.9;19.7]	14.8 [11.8;19.6]	14.8 [11.9;20.3]	0.777
CKMB, Median [IQR], ng/mL	2.3 [1.5;3.8]	2.3 [1.5;3.7]	2.3 [1.5;4.3]	0.996
BNP, Median [IQR], pg/mL	231.5 [99.0;406.5]	222.0 [93.0;398.0]	249.0 [125.0;412.0]	0.071
LDH, Median [IQR], U/L	187.0 [160.0;234.0]	185.0 [159.0;234.0]	188.5 [162.0;232.8]	0.594
cTnI, Median [IQR], ng/mL	0.1 [<0.1;0.8]	0.1 [<0.1;0.7]	0.1 [<0.1;0.9]	0.669
Sodium, mean (±SD), mmol/L	139.0 ± 3.0	139.0 ± 3.0	139.1 ± 3.1	0.613
Potassium, mean (±SD), mmol/L	4.2 ± 0.4	4.2 ± 0.4	4.1 ± 0.4	0.566
Chloride, mean (±SD), mmol/L	102.4 ± 3.2	102.5 ± 3.2	102.3 ± 3.1	0.398
Calcium, mean (±SD), mmol/L	2.3 ± 0.1	2.3 ± 0.1	2.3 ± 0.1	0.353
Magnesium, mean (±SD), mmol/L	0.9 ± 0.1	0.9 ± 0.1	0.9 ± 0.1	0.830
PT, mean (±SD), s	12.0 ± 3.4	12.0 ± 3.3	12.1 ± 3.7	0.902
APTT, Median [IQR], s	31.0 [29.0;33.0]	31.0 [29.0;33.0]	31.0 [28.0;32.0]	0.208
INR, Median [IQR]	1.0 [1.0;1.1]	1.0 [1.0;1.1]	1.0 [1.0;1.1]	0.448
FDP, Median [IQR], μg/mL	0.8 [0.4;1.5]	0.8 [0.4;1.4]	0.8 [0.4;1.7]	0.163
FBG, Median [IQR], g/L	3.3 [2.9;3.9]	3.3 [2.9;3.9]	3.3 [3.0;4.0]	0.369
D-dimer, Median [IQR], ng/L	127.0 [83.0;215.8]	126.0 [80.0;207.0]	133.0 [87.0;244.0]	0.054
Coronary artery lesions				
Prior MI, *N* (%)	238 (18.6%)	175 (18.9%)	63 (17.6%)	0.656
Prior PCI, *N* (%)	127 (9.9%)	98 (10.6%)	29 (8.1%)	0.221
UA, *N* (%)	754 (58.8%)	548 (59.2%)	206 (57.7%)	0.661
STEMI, *N* (%)	17 (1.3%)	14 (1.5%)	3 (0.8%)	0.426
NSTEMI, *N* (%)	123 (9.6%)	89 (9.6%)	34 (9.5%)	0.999
SYNTAX, mean (±SD)	21.8 ± 7.8	21.6 ± 7.7	22.5 ± 8.0	0.060
Target LM, *N* (%)	217 (16.9%)	150 (16.2%)	67 (18.8%)	0.313
Target LAD, *N* (%)	941 (73.4%)	666 (72.0%)	275 (77.0%)	0.079
Target LCX, *N* (%)	826 (64.4%)	599 (64.8%)	227 (63.6%)	0.743
Target RCA, *N* (%)	897 (70.0%)	652 (70.5%)	245 (68.6%)	0.560
Stent number, Median [IQR]	3.0 [2.0;4.0]	3.0 [2.0;4.0]	3.0 [2.0;4.0]	0.075
Long stent, *N* (%)	687 (53.6%)	489 (52.9%)	198 (55.5%)	0.439
Total revascularization, *N* (%)	799 (62.3%)	574 (62.1%)	225 (63.0%)	0.797
ISR, *N* (%)	65 (5.1%)	54 (5.8%)	11 (3.1%)	0.061
CTO, *N* (%)	367 (28.6%)	256 (27.7%)	111 (31.1%)	0.253
Ostial lesion, *N* (%)	117 (9.1%)	88 (9.5%)	29 (8.1%)	0.505
Bifurcation, *N* (%)	181 (14.1%)	124 (13.4%)	57 (16.0%)	0.275
Tortuous lesion, *N* (%)	58 (4.5%)	37 (4.0%)	21 (5.9%)	0.192
Calcification, *N* (%)	202 (15.8%)	137 (14.8%)	65 (18.2%)	0.158
Diffuse lesion, *N* (%)	262 (20.4%)	179 (19.4%)	83 (23.2%)	0.140
Medication				
Aspirin, *N* (%)	1,272 (99.2%)	917 (99.1%)	355 (99.4%)	0.735
Clopidogrel, *N* (%)	1,054 (82.2%)	750 (81.1%)	304 (85.2%)	0.104
Ticagrelor, *N* (%)	228 (17.8%)	175 (18.9%)	53 (14.8%)	0.104
Statins, N (%)	1,267 (98.8%)	916 (99.0%)	351 (98.3%)	0.383
ACEI, *N* (%)	128 (10.0%)	96 (10.4%)	32 (9.0%)	0.513
ARB, *N* (%)	192 (15.0%)	123 (13.3%)	69 (19.3%)	0.009
Sacubitril valsartan, *N* (%)	419 (32.7%)	320 (34.6%)	99 (27.7%)	0.022
Beta blocker, *N* (%)	741 (57.8%)	534 (57.7%)	207 (58.0%)	0.985
Nitrates, *N* (%)	1,175 (91.7%)	844 (91.2%)	331 (92.7%)	0.458

For continuous variables, data following a normal distribution were expressed as mean ± standard deviation (SD) and compared using the independent samples *t*-test. Non-normally distributed data were presented as median [interquartile range, IQR] and compared using the Mann–Whitney *U*-test. For comparisons among more than two groups, one-way analysis of variance (ANOVA) was used for normally distributed variables, and the Kruskal–Wallis *H*-test was applied for non-normally distributed variables. Categorical variables were presented as numbers and percentages and analyzed using the Chi-square test or Fisher's exact test, as appropriate. A two-tailed *P* value < 0.05 was considered statistically significant. SBP, systolic blood pressure; DBP, diastolic blood pressure; BMI, body mass index; DM, diabetes mellitus; WBC, white blood cell; RBC, red blood cell; ALT, alanine aminotransferase; AST, aspartate aminotransferase; ALP alkaline phosphatase; HbA1c, hemoglobin A1c; EGFR, estimated glomerular filtration rate; TC, total cholesterol; LDL-c, low-density lipoprotein cholesterol; HDL-c, high-density lipoprotein cholesterol; hs-CRP, high-sensitivity C-reactive protein; CKMB, creatine kinase-MB; BNP, B-type natriuretic peptide; LDH, lactate dehydrogenase; cTnI, cardiac troponin I; PT, prothrombin time; APTT, activated partial thromboplastin time; INR, international normalized ratio; FDP, fibrin degradation products; FBG, fasting blood glucose; MI, myocardial infarction; PCI, percutaneous coronary intervention; UA, unstable angina; STEMI, ST-segment elevation myocardial infarction; NSTEMI, non-ST-segment elevation myocardial infarction; SYNTAX, synergy between PCI with taxus and cardiac surgery score; LM, left main; LAD, left anterior descending artery; LCX, left circumflex artery; RCA, right coronary artery; ISR, in-stent restenosis; CTO, chronic total occlusion; ACEI, angiotensin-converting enzyme inhibitor; ARB, angiotensin receptor blocker.

**Table 2:** Distribution of intrinsic capacity in different outcomes.

Variables	Total	No-rehospitalization	Rehospitalization	*P*
Intrinsic capacity score, Median [IQR]	1.0 [0.0;2.0]	1.0 [0.0;2.0]	2.0 [1.0;2.0]	<0.001
The psychological domain				
Exhaustion, *N* (%)	171 (13.3%)	123 (13.3%)	48 (13.4%)	0.999
Sleep duration, *N* (%)	343 (26.8%)	238 (25.7%)	105 (29.4%)	0.206
The sensory domain				
Vision impairment, *N* (%)	67 (5.2%)	35 (3.8%)	32 (9.0%)	<0.001
Hearing difficulty, *N* (%)	337 (26.3%)	144 (15.6%)	193 (54.1%)	<0.001
The vitality domain				
Declined grip strength, *N* (%)	253 (19.7%)	163 (17.6%)	90 (25.2%)	0.003
Weight loss, *N* (%)	111 (8.7%)	74 (8.0%)	37 (10.4%)	0.216
Walking difficulty, *N* (%)	211 (16.5%)	149 (16.1%)	62 (17.4%)	0.645

**Table 3:** Distribution of intrinsic capacity in different gender.

Variables	Total	Male	Female	*P*
Intrinsic capacity score, Median [IQR]	1.0 [0.0;2.0]	1.0 [0.0;2.0]	1.0 [0.0;2.0]	0.214
The psychological domain				
Exhaustion, *N* (%)	171 (13.3%)	124 (12.9%)	47 (14.5%)	0.535
Sleep duration, *N* (%)	343 (26.8%)	264 (27.6%)	79 (24.4%)	0.297
The sensory domain				
Vision impairment, *N* (%)	67 (5.2%)	52 (5.4%)	15 (4.6%)	0.679
Hearing difficulty, *N* (%)	337 (26.3%)	262 (27.3%)	75 (23.1%)	0.158
The vitality domain				
Declined grip strength, *N* (%)	253 (19.7%)	191 (19.9%)	62 (19.1%)	0.816
Weight loss, *N* (%)	111 (8.7%)	85 (8.9%)	26 (8.0%)	0.723
Walking difficulty, *N* (%)	211 (16.5%)	153 (16.0%)	58 (17.9%)	0.469

We use the Cox regression analysis to examine the association between IC score and different types of rehospitalization (all-cause rehospitalization, non-cardiac rehospitalization, and cardiac rehospitalization (Table 4). For all-cause rehospitalization, in the crude model, higher levels of IC score were associated with increased HRs. Individuals with IC score = 2 had a significantly higher risk of rehospitalization (HR = 2.72, 95% CI: 2.02–3.64, *P* < 0.001), while those with IC score = 3 and ≥4 showed even higher risks (HR = 3.42, 95% CI: 2.41–4.85, *P* < 0.001 and HR = 5.34, 95% CI: 3.37–8.44, *P* < 0.001, respectively). After adjusting for confounders in the partially adjusted model, IC score = 3 and ≥4 remained significant. In the full-adjusted model, IC score = 3 and ≥4 still had significant associations with rehospitalization (HR = 2.18, 95% CI: 1.53–3.12, *P* < 0.001, and HR = 3.07, 95% CI: 1.89–5.00, *P* < 0.001). For non-cardiac rehospitalization, in the fully adjusted model, only IC score ≥4 remained a significant predictor of non-cardiac rehospitalization (HR = 2.20, 95% CI: 1.17–4.15, *P* = 0.015), but IC score = 2 and 3 were no longer significant. For cardiac rehospitalization, individuals with higher IC score showed much stronger associations in the crude model. After partial adjustment, these associations persisted. In the full-adjusted model, the associations still remained significant for IC score =2,3, and ≥4. (HR = 2.64, 95% CI: 1.50–4.64, *P* = 0.001; HR = 4.65, 95% CI: 2.53–8.54, *P* < 0.001; and HR = 5.23, 95% CI: 2.30–11.89, *P* < 0.001, respectively. IC score is strongly associated with the risk of rehospitalization, particularly for cardiac rehospitalization after full adjustment for confounding factors.

**Table 4:** Cox regression analysis of rehospitalization according to IC score.

Variables	Crude model			Partially adjusted model			Full-adjusted model		
	HR	95% CI	*P*	HR	95% CI	*P*	HR	95% CI	*P*
All-cause rehospitalization									
IC score = 0	ref	ref	ref	ref	ref	ref	ref	ref	ref
IC score = 1	1.11	0.82–1.50	0.504	1.05	0.78–1.42	0.734	1.04	0.77–1.41	0.806
IC score = 2	2.72	2.02–3.64	<0.001	1.37	1.01–1.86	0.046	1.33	0.98–1.81	0.072
IC score = 3	3.42	2.41–4.85	<0.001	2.09	1.46–2.98	<0.001	2.18	1.53–3.12	<0.001
IC score ≥ 4	5.34	3.37–8.44	<0.001	2.88	1.80–4.61	<0.001	3.07	1.89–5.00	<0.001
IC (continuous)	1.60	1.46–1.75	<0.001	1.30	1.18–1.43	<0.001	1.30	1.18–1.44	<0.001
Non-Cardiac rehospitalization									
IC score = 0	ref	ref	ref	ref	ref	ref	ref	ref	ref
IC score = 1	1.05	0.78–1.42	0.734	1.06	0.75–1.49	0.743	1.04	0.74–1.47	0.806
IC score = 2	1.37	1.01–1.86	0.046	0.95	0.65–1.39	0.798	0.92	0.63–1.35	0.665
IC score = 3	2.09	1.46–2.98	<0.001	1.31	0.82–2.11	0.255	1.38	0.86–2.22	0.179
IC score ≥ 4	2.88	1.80–4.61	<0.001	2.13	1.15–3.93	0.016	2.20	1.17–4.15	0.015
IC (continuous)	1.39	1.24–1.56	<0.001	1.11	0.98–1.26	0.098	1.11	0.97–1.26	0.116
Cardiac rehospitalization									
IC score = 0	ref	ref	ref	ref	ref	ref	ref	ref	ref
IC score = 1	1.10	0.59–2.05	0.771	1.04	0.56–1.94	0.904	1.03	0.55–1.93	0.928
IC score = 2	5.20	3.03–8.92	<0.001	2.69	1.53–4.72	0.001	2.64	1.50–4.64	0.001
IC score = 3	7.25	4.01–13.12	<0.001	4.53	2.47–8.30	<0.001	4.65	2.53–8.54	<0.001
IC score ≥ 4	8.82	4.07–19.14	<0.001	4.71	2.13–10.44	<0.001	5.23	2.30–11.89	<0.001
IC (continuous)	1.98	1.71–2.28	<0.001	1.64	1.40–1.92	<0.001	1.66	1.41–1.95	<0.001

Crude model was unadjusted. Partially adjusted model was adjusted for age and sex. Full adjusted model was adjusted for age, sex, stent number, tortuous lesion, target LCX, hypertension, monocytes. HR, hazard ratio; CI, confidence interval; IC, intrinsic capacity.

**Figure 2:**
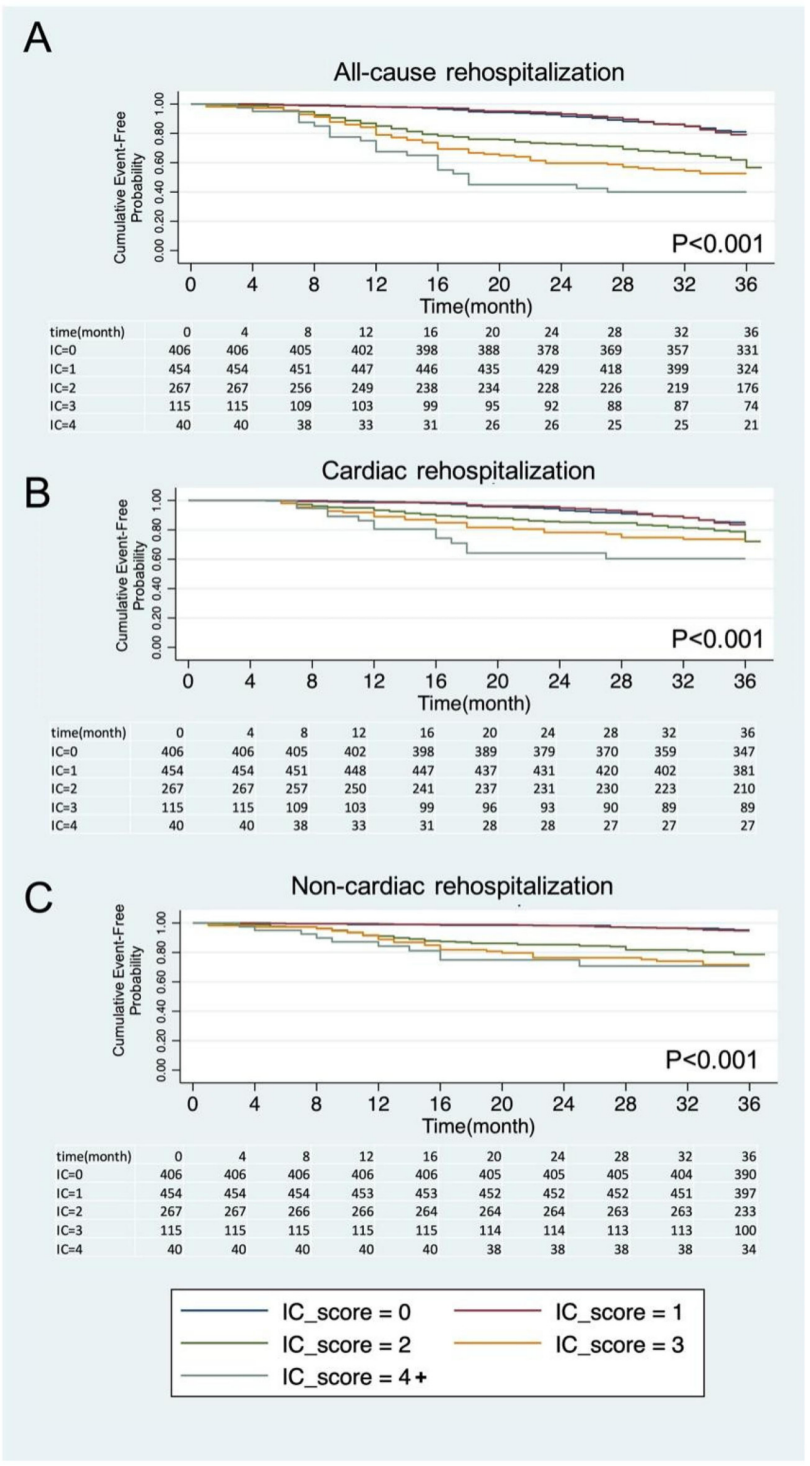
Kaplan–Meier (KM) survival curves for all-cause rehospitalization **(A)**, cardiac rehospitalization **(B)**, and non-cardiac rehospitalization **(C)** based on intrinsic capacity score (IC) levels. IC, intrinsic capacity.

The survival curves illustrated the incidence of rehospitalization over time for different levels of IC score. In all-cause rehospitalization, higher intrinsic IC score groups (IC ≥ 2) are associated with a higher incidence of rehospitalization compared with IC score =0 and 1 group. In non-cardiac rehospitalization, similar trends are observed, with individuals in the higher IC score groups (≥2) having a higher risk of non-cardiac rehospitalization. In terms of cardiac rehospitalization, higher IC score group (≥2) also had the higher rates of cardiac rehospitalization, compared to those with lower IC values (log-rank test all *P* < 0.001, Figure 2).

**Figure 3:**
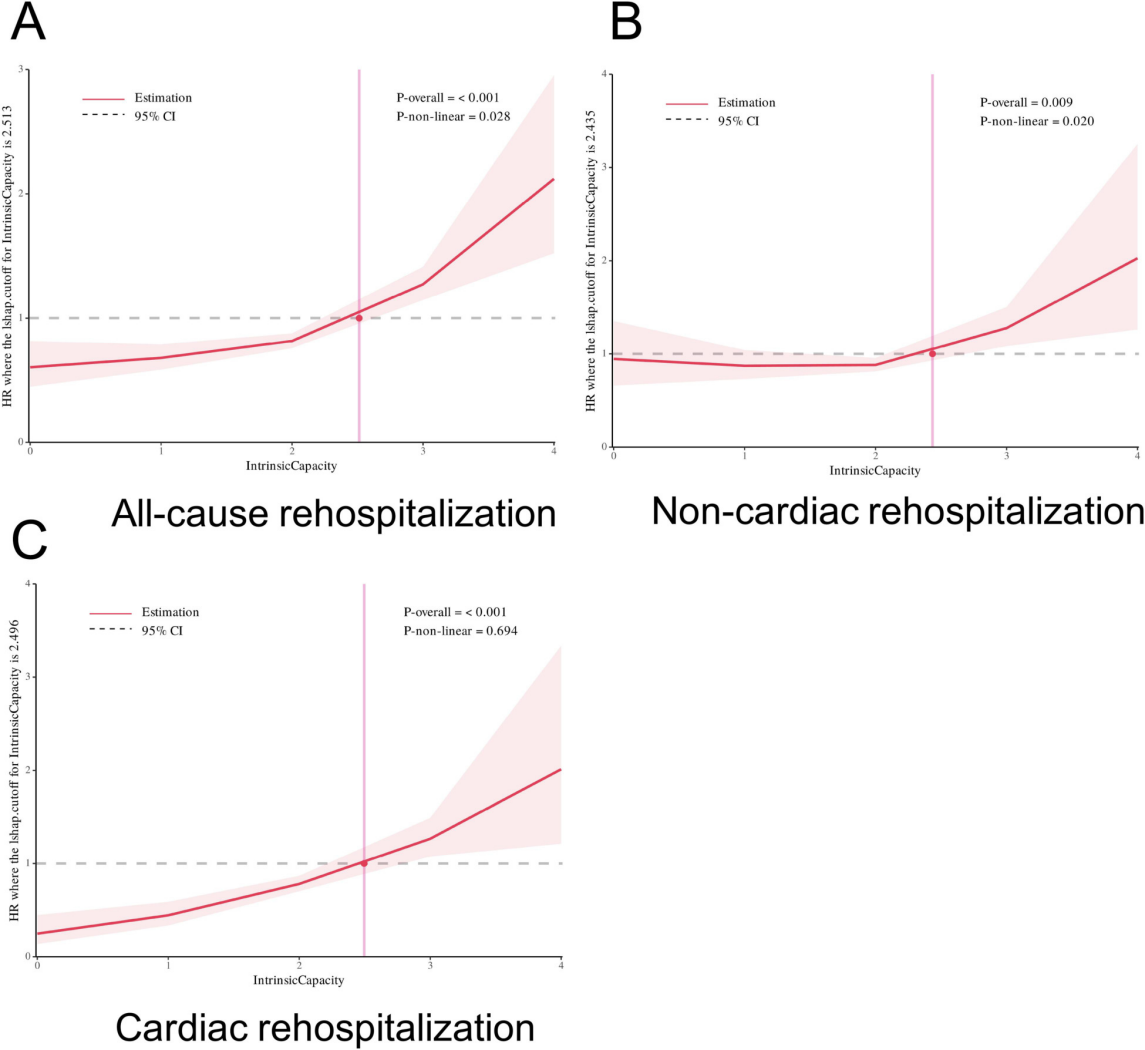
Restricted cubic spline (RCS) curves illustrating the association between intrinsic capacity (IC) and the hazard ratios for all-cause rehospitalization **(A)**, non-cardiac rehospitalization **(B)**, and cardiac rehospitalization **(C)**. The shaded areas represent 95% confidence intervals for the estimates. The cutoff value of IC score was 2.5. HR, hazard ratio; CI, confidence interval.

The RCS curves illustrate the relationship between IC score and the HRs for all-cause rehospitalization, non-cardiac rehospitalization, and cardiac rehospitalization. In all-cause rehospitalization (Figure 3A), there is a positive non-linear association between IC and rehospitalization, with the HR increasing drastically for IC values greater than 2 (P for trend <0.001 and P for non-linear = 0.028). Figure 3B (Non-cardiac rehospitalization) showed a similar pattern, where the slope of HRs elevated with increasing IC score, particularly beyond IC = 2.5(P for trend = 0.009 and P for non-linear = 0.020). In Figure 3C (Cardiac rehospitalization), the HR increases for higher IC levels, with a highly significant overall association (*P* < 0.001), although the non-linear association is not significant (*P* = 0.694).

**Figure 4:**
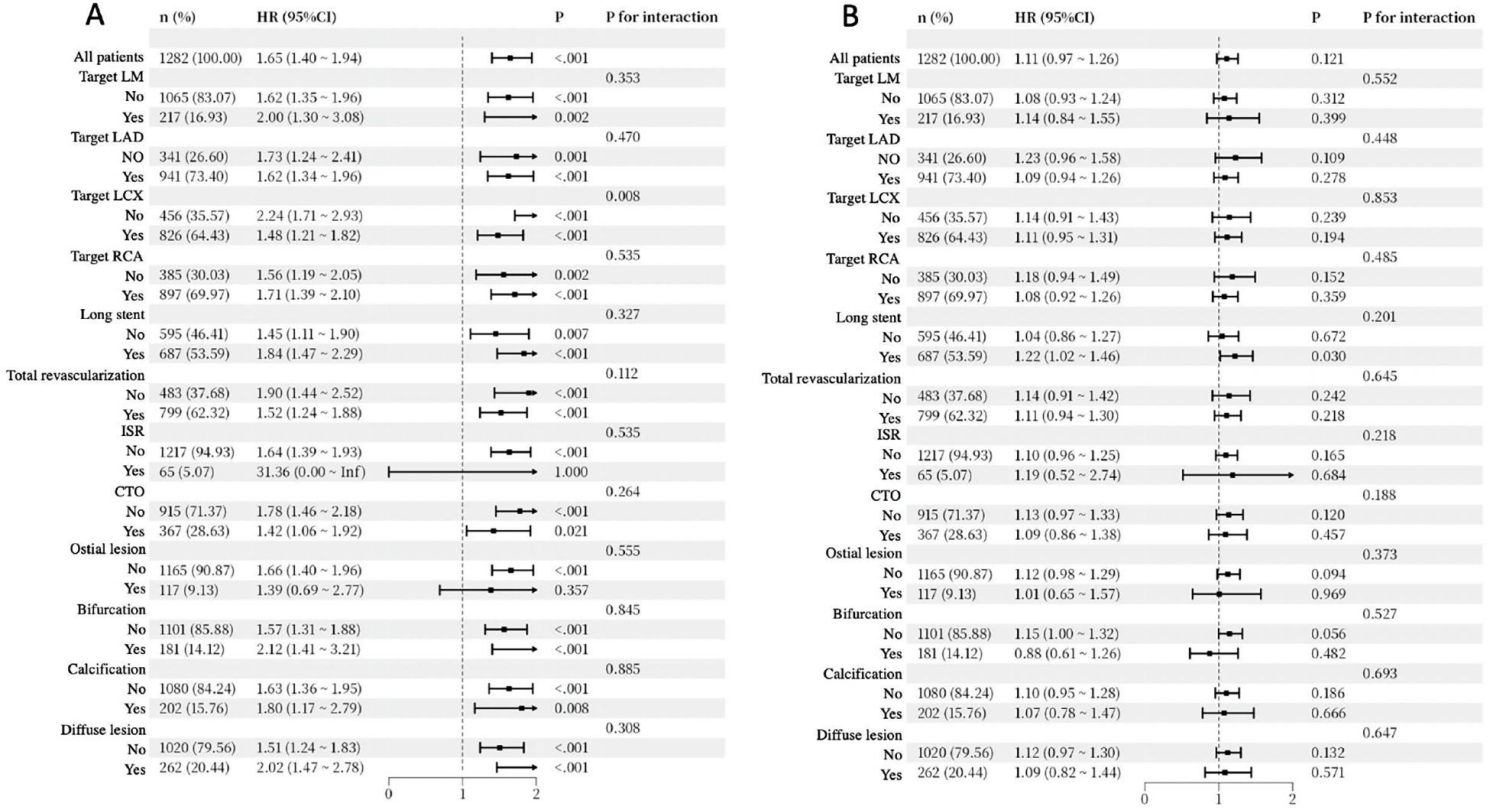
Subgroup analysis and forest plots for cardiac rehospitalization **(A)** and non-cardiac rehospitalization **(B)** LM, left main (coronary artery); LAD, left anterior descending artery; LCX, left circumflex artery; RCA, right coronary artery; HR, hazard ratio; CI, confidence interval; ISR, in-stent restenosis; CTO, chronic total occlusion.

To further explore the relationship between IC and lesion characteristics or revascularization status, we carried out the subgroup analysis of cardiac rehospitalization and non-cardiac rehospitalization for different subgroups. Figure 4A (cardiac rehospitalization) demonstrated that the association between IC and clinical outcomes remained largely consistent across most clinical and procedural categories. No significant interactions were observed for factors such as target lesions (LM, LAD, RCA), stenting characteristics, and lesion complexity (all P for interaction > 0.05). Notably, a significant interaction was found for Target LCX (all P for interaction = 0.008), where the predictive value of IC was more pronounced in patients without LCX lesions (HR: 2.24; 95% CI: 1.71–2.93) compared to those with LCX lesions (HR: 1.48; 95% CI: 1.21–1.82). Despite this specific variation, the overall results suggest that the prognostic value of IC is robust across diverse clinical settings, with no significant heterogeneity observed in the vast majority of subgroups. On the contrary, Subgroup analysis for non-cardiac rehospitalization (Figure 4B) indicated no significant predictive value for non-cardiac rehospitalization in the overall population (HR: 1.11, 95% CI: 0.97–1.26, *P* = 0.121). Subgroup analyses further confirmed the uniformity of this lack of association, as no significant interactions were observed across all clinical and procedural categories (all P for interaction > 0.05). While the Long stent subgroup reached significance (HR: 1.22, 95% CI: 1.02–1.46, *P* = 0.030), the corresponding interaction test remained non-significant (P for interaction = 0.201), which should be interpreted as consistent with the overall null effect. These suggest that the predictive ability of the IC score for non-cardiac rehospitalization is limited, as it does not consistently predict rehospitalization across most subgroups.

**Figure 5:**
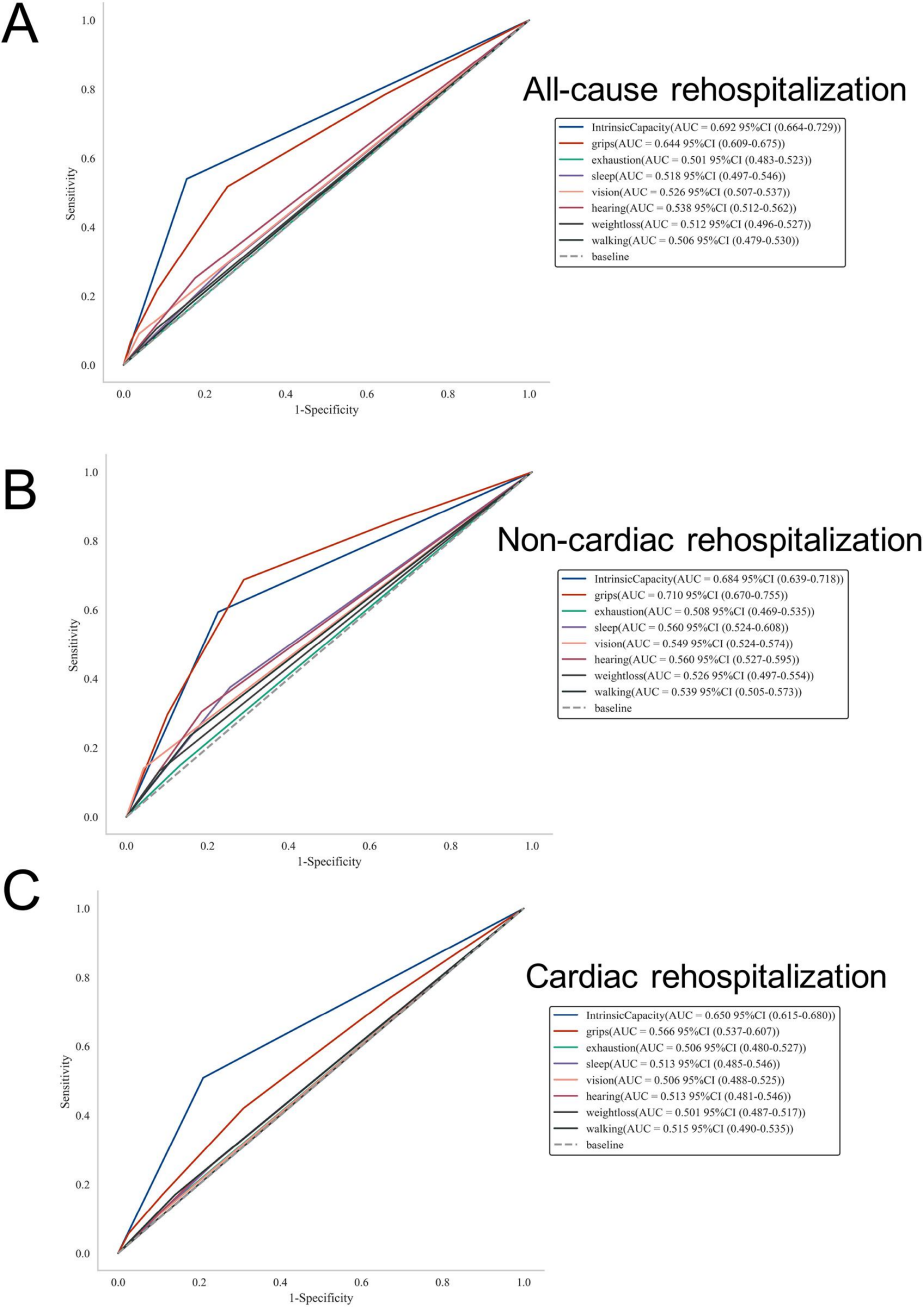
Receiver operating characteristic (ROC) curves assessing the predictive performance of intrinsic capacity score and its components for rehospitalization outcomes. **(A)** The ROC curve for all-cause rehospitalization; **(B)** the ROC curve for non-cardiac rehospitalization, **(C)** the ROC curve for cardiac rehospitalization. ROC, receiver operating characteristic; AUC, area under curve; HR, hazard ratio; CI, confidence interval.

Lastly, we examined the predictive efficacy of IC score and its components (including exhaustion, sleep duration, vision impairment, hearing difficulty, declined grip strength, weight loss, walking difficulty) in different types of rehospitalization outcomes. Figure 5A displays the ROC curve for all-cause rehospitalization, with IC score showing the highest AUC of 0.692 (95% CI: 0.664–0.729), indicating moderate predictive performance. Figure 5B shows the ROC curve for non-cardiac rehospitalization, where IC score achieves an AUC of 0.684 (95% CI: 0.639–0.718). Declined grip strength showed the highest AUC of 0.710. In Figure 5C, the ROC curve for cardiac rehospitalization is presented with IC score having an AUC of 0.650 (95% CI: 0.615–0.680). Besides, the model showed acceptable discrimination, with a C-index of 0.67 for the total IC score. The C-indices for individual IC components ranged from 0.50 to 0.65 (Detailed metrics: hand grip 0.65, hearing 0.55, sleep 0.53, vision 0.52, weight loss 0.51, walking pace 0.51, and exhaustion 0.50).

## Discussion

A substantial proportion of post-PCI CAD patients experience recurrent hospitalizations, which negatively impact prognosis and quality of life. Identifying reliable predictors of post-PCI readmission remains a clinical priority (3). To our knowledge, this is the first study to demonstrate that IC is an independent predictor of cardiovascular-related rehospitalization in this population. Notably, we observed that IC had greater predictive value for cardiac readmission than for non-cardiac causes. Furthermore, this study is also the first to explore the relationship between IC and target vessel lesion morphology as well as interventional outcomes. Subgroup analyses confirmed that the prognostic value of IC was consistent across lesion morphologies and revascularization strategies, suggesting that IC is a reliable predictor.

The underlying mechanisms through which IC predicts cardiac rehospitalization may be multifactorial. First, IC encompasses psychological function, sensory function, and vitality function, which are strongly associated with adherence to therapy, symptom recognition, lifestyle behaviors, and timely healthcare engagement (18). Declines in IC may indicate poor exercise tolerance, low resilience to stressors, reduced self-management capacity, and diminished physiologic reserve. These are known contributors to cardiovascular decompensation and adverse outcomes (19). Second, patients with reduced IC may experience subclinical decline or subtle manifestations of recurrent ischemia or heart failure that are poorly self-reported or under-recognized until decompensation prompts readmission (20). The finding that IC had relatively limited predictive value for non-CV readmissions supports the notion that IC is more closely linked to disease-specific vulnerability in cardiovascular physiology (21).

Handgrip strength in our study was found as a significant prognostic marker for non-cardiac rehospitalization, where its discriminative ability was statistically comparable to that of the full IC score. Grip strength has been consistently associated with outcomes such as hospitalization, disability, and mortality in diverse populations (22). In the large-scale population-based study showed that grip strength is closely related with the specific-cause mortality of all respiratory disease and all cancer (23, 24). Besides, the study also finds that low handgrip strength is also associated with functional limitations and disability. These findings are consistent with our study (25). The underlying mechanism might be that Grip strength reflects nutrition deprivation, physiological frailty, and global functional decline, making it more sensitive to predicting non-cardiovascular readmissions, such as infections, falls, or decompensation of chronic conditions, which are closely linked to reduced muscular strength and overall physical resilience (24, 26–28). However, handgrip showed less effective in predicting cardiac rehospitalization, which in contradictive with previous studies (29, 30). Several previous studies have demonstrated a strong association between reduced handgrip strength and poor prognosis in patients with CAD (31). The increase in grip strength was associated with reduction in mortality and cardiovascular event risk (32). Additionally, grip strength effectively predicted exercise capacity in CAD patients (33–35). In our study, the reason why handgrip strength can't predict cardiac rehospitalization properly is that grip strength may be more sensitive to the prediction of primary events rather than recurrent events, which are often influenced by a broader range of clinical and procedural factors (36). Second, in our cohort, the relatively limited number of cardiovascular readmission events may have reduced the statistical power to detect a significant association. Given all these findings, grip strength may serve as a quick pre-screening for general resilience, particularly in settings where full IC assessment is not feasible (37).

In the field of chronic disease management, apart from IC score, the frailty index (FI) and clinical frailty scale (CFS) are widely used to assess vulnerability in older adults. Just like IC score, these instruments are also grounded in a deficit accumulation model (38). Studies have shown that both FI and CFS are predictive of mortality, hospitalizations, and complications in cardiovascular patients (38, 39). However, these tools are often complex, require detailed data input, and are predominantly used in geriatric or perioperative settings. In contrast, IC score represents a capability-based approach that evaluates an individual's physical and mental reserves (40). Importantly, the IC score is practical and easy to implement in clinical settings. It can be rapidly assessed upon hospital admission using a structured questionnaire that does not rely on specialized diagnostic procedures and can be administered by trained nurses within 15 min, making it a feasible tool for routine screening and early risk stratification (41). Furthermore, IC assessment by nursing staff allows for more targeted discharge education. For patients identified at higher risk of readmission, especially for cardiovascular causes, nurses can place greater emphasis on symptom recognition, medication adherence, and timely medical follow-up, potentially improving post-discharge outcomes (42).

To effectively mitigate the risk of rehospitalization in PCI-CAD patients, postoperative management should transition from a disease-centered approach toward a comprehensive intervention framework focused on IC. Evidence indicates that all five domains of IC—locomotion, vitality, cognition, psychological, and sensory—serve as critical targets for intervention (43–45). First, multi-component cardiac rehabilitation programs, incorporating resistance, balance, and moderate-intensity aerobic training, should be implemented to enhance locomotor capacity and counteract sarcopenia (46, 47). Second, in the vitality domain, adopting a Mediterranean diet and optimizing metabolic reserves through high-quality protein and fiber intake is recommended (48). Third, to safeguard cognitive and psychological capacities, routine screening Montreal Cognitive Assessment (MoCA) should be used in older patients. Concurrently, integrating cognitive behavioral therapy (CBT) can alleviate postoperative anxiety and depression, thereby strengthening psychological resilience and self-management (49). Finally, sensory compensation through the provision of hearing aids and corrective lenses, alongside sensory-friendly health education, forms the foundation for effective secondary prevention (50). Utilizing the WHO ICOPE management framework enables clinical-community-family synergy for early screening and personalized intervention, ultimately increasing functional reserves and reducing unplanned rehospitalizations (51, 52).

Despite its strengths, this study has several limitations. First, one major limitation of this study is single-center design and the absence with internal or external validation, which restricts the generalizability of the findings, making it likely that the model's performance may not be applicable to broader, more diverse populations. Second, while the IC score is practical and reproducible, some domains (e.g., cognition or sensory function) may be under- or over-estimated in the absence of objective testing. Third, another limitation of this study is that a small number of patients experienced sudden unexplained deaths outside the hospital during the follow-up period. These cases were not classified as readmissions due to the absence of hospitalization records or definitive diagnostic information. Although the number of such events was limited, they may reflect severe underlying cardiovascular events and introduce a potential source of bias, potentially underestimating the true burden of adverse outcomes in this population. Future studies should aim to incorporate more comprehensive follow-up strategies to better capture out-of-hospital events. Additionally, prospective multicenter studies with longer follow-up periods and adjudicated outcomes would help validate the prognostic value of IC.

## Conclusion

In this retrospective cohort study of patients with CAD undergoing PCI, IC was identified as an independent predictor of cardiovascular rehospitalization. Its predictive value remained consistent across various lesion types and procedural strategies. IC assessment is a practical and reliable tool for post-discharge planning in high-risk cardiac populations.

## Data Availability

The raw data supporting the conclusions of this article will be made available by the authors, without undue reservation.
