# Supplementary material for: Early nurse-assessed intrinsic capacity stratifies rehospitalization risk after percutaneous coronary intervention in coronary artery disease patients
Source: Front Cardiovasc Med. 2026 Apr 20;13:1763248. doi: 10.3389/fcvm.2026.1763248 (PMC13135967; doi:10.3389/fcvm.2026.1763248)
Supplement: Supplementary file 1 [file Table1.docx]

Statistical analysis

Continuous variables were presented as mean ± standard deviation (SD) for normally distributed data, or as median and interquartile range (IQR) for non-normally distributed data. Categorical variables were expressed as counts and percentages. Categorical variables had no missing data. Continuous variables were deleted if missing data exceeded 10%; otherwise, missing values were filled using multiple imputation. Between-group comparisons were performed using the independent-samples t-test or Mann–Whitney U test for continuous variables, and the chi-square test or Fisher’s exact test for categorical variables, as appropriate. To identify potential confounders, we use a stepwise backward elimination method with a threshold of P < 0.05 for retention in the multivariable model. Cox proportional hazards regression models were used to estimate hazard ratios (HRs) and 95% confidence intervals (CIs) for the association between IC score and the risk of rehospitalization, including all-cause, cardiac, and non-cardiac rehospitalization. Kaplan–Meier survival curves were generated to compare event-free survival across IC score categories, and the log-rank test was used to assess statistical significance. Restricted cubic spline (RCS) analysis was conducted to explore potential non-linear relationships between IC score and the risk of different types of hospitalization with knots placed at predefined percentiles of the IC score distribution. Subgroup analyses were performed to assess the consistency of the association between IC and outcomes across clinically status. All subgroup analyses were pre-specified based on clinical relevance. The results were visualized using forest plots. Receiver operating characteristic (ROC) curve analysis was used to evaluate the discriminatory ability of IC and its components for predicting rehospitalization. The area under the curve (AUC) and corresponding 95% CIs were reported for comparison. All statistical analyses were performed using STATA (MP 17) and R software (version 4.2.1, R Foundation for Statistical Computing, Vienna, Austria). The following R packages were used: survival, survminer, rms, forestplot, pROC. A two-tailed P value < 0.05 was considered statistically significant.

Intrinsic capacity investigation（English version）

patient ID： patient name：

investigator： study date：

verifier： verifying date：

| **PART 1 THE PSYCHOLOGICAL DOMAIN** |
| --- |
| Exhaustion |
| Q1: Over the past two weeks, how often have you felt tired or had little energy? |
| R1_1: more than half of the days, nearly every day. □ 1 point |
| R1_2: not at all/several days. □ 0 point |
| C1: other comments_________________ |
| Sleep duration |
| Q2: About how many hours sleep do you get in every 24 hours? please include naps |
| R2_1: short <7 h/day or long >9 h/day. □ 1 point |
| R2_2: healthy 7–9 h/day. □ 0 point |
| C2: other comments_________________ |
| **PART 2 THE SENSORY DOMAIN** |
| Vision impairment |
| Q3: Do you have considering eye/eyelid problems (non-cancer illness) |
| R3_1: yes, I can’t see anything clearly. □ 1 point |
| R3_2: no. □ 0 point |
| C3: other comments_________________ |
| Hearing difficulty |
| Q4: Do you have any difficulty with your hearing? |
| R4_1: yes, I am completely deaf. □ 1 point |
| R4_2: no. □ 0 point |
| C4: other comments_________________ |
| **PART3 THE VITALITY DOMAIN** |
| Q5: Declined grip strength |
| R5_1: below handgrip strength cut-off value □ 1 point |
| R5_2: above handgrip strength cut-off value □ 0 point |
| C5: other comments_________________ |
| grip strength was assessed by hydraulic hand dynamometer (Jamar J00105), and the mean of the right and left values was expressed in absolute units (kilograms), and by sex and body mass index adjusted |
| Weight loss |
| Q6: Compared with one year ago, has your weight changed? |
| R6_1: yes, I lost weight. □ 1 point |
| R6_2: no, I weight the same. □ 0 point |
| R6_3: yes, I gain weight. □ 0 point |
| C6: other comments_________________ |
| Walking difficulty |
| Q7: How would you describe your usual walking pace? |
| R7_1: slow pace. □ 1 point |
| R7_2: steady average pace or brisk pace. □ 0 point |
| C7: other comments_________________ |
| **TOTAL POINT：** |
